# Genome-Wide Association Study and Genomic Prediction of Soybean Mosaic Virus Resistance

**DOI:** 10.3390/ijms26052106

**Published:** 2025-02-27

**Authors:** Di He, Xintong Wu, Zhi Liu, Qing Yang, Xiaolei Shi, Qijian Song, Ainong Shi, Dexiao Li, Long Yan

**Affiliations:** 1 Institute of Cereal and Oil Crops, Hebei Academy of Agricultural and Forestry Sciences, Shijiazhuang 050035, China; ternuraeyes@163.com (D.H.); xintongwu66@163.com (X.W.); zhiliulin@sina.com (Z.L.); qyang0807008@163.com (Q.Y.); shixiaolei59@163.com (X.S.); 2 College of Life Sciences, Hebei Agricultural University, Baoding 071001, China; 3 Soybean Genomics and Improvement Laboratory, Agricultural Research Service, Beltsville, MD 20705, USA; qijian.song@usda.gov; 4 Department of Horticulture, University of Arkansas, Fayetteville, AR 72701, USA; ashi@uark.edu; 5 College of Agronomy, Northwest A&F University, Yangling 712100, China

**Keywords:** soybean mosaic virus (SMV), genome-wide association study (GWAS), genomic prediction (GP), quantitative trait locus (QTL), marker-assisted selection (MAS)

## Abstract

Soybean mosaic virus (SMV), a pathogen responsible for inducing leaf mosaic or necrosis symptoms, significantly compromises soybean seed yield and quality. According to the classification system in the United States, SMV is categorized into seven distinct strains (G1 to G7). In this study, we performed a genome-wide association study (GWAS) in GAPIT3 using four analytical models (MLM, MLMM, FarmCPU, and BLINK) on 218 soybean accessions. We identified 22 SNPs significantly associated with G1 resistance across chromosomes 1, 2, 3, 12, 13, 17, and 18. Notably, a major quantitative trait locus (QTL) spanning 873 kb (29.85–30.73 Mb) on chromosome 13 exhibited strong association with SMV G1 resistance, including the four key SNP markers: Gm13_29459954_ss715614803, Gm13_29751552_ss715614847, Gm13_30293949_ss715614951, and Gm13_30724301_ss715615024. Within this QTL, four candidate genes were identified: *Glyma.13G194100*, *Glyma.13G184800*, *Glyma.13G184900*, and *Glyma.13G190800* (*3Gg2*). The genomic prediction (GP) accuracies ranged from 0.60 to 0.83 across three GWAS-derived SNP sets using five models, demonstrating the feasibility of GP for SMV-G1 resistance. These findings could provide a useful reference in soybean breeding targeting SMV-G1 resistance.

## 1. Introduction

Soybean (*Glycine max* (L.) Merr.), a cultivated oilseed crop, serves as a vital source of plant-based protein and edible oil, contributing substantially to agricultural economies worldwide [1]. However, soybean productivity is severely compromised by soybean mosaic virus (SMV), a member of the Potyvirus genus that induces characteristic symptoms such as leaf mosaic, localized necrosis at infection sites, systemic necrosis vein browning, and apical necrosis [2].

In the United States, SMV isolates are classified into seven distinct strains (G1–G7) based on differential interactions with soybean genotypes [3]. Four major SMV resistance loci (*Rsv1*, *Rsv3*, *Rsv4*, and *Rsv5*) have been identified across genetic studies [2,4,5]. *Rsv1*, located on chromosome 13, which encodes a cluster of nucleotide-binding leucine-rich repeat (NLR) proteins conferring resistance to G1–G6 strains, with multiple allelic variants (*Rsv1*, *Rsv1-t*, *Rsv1-m*, *Rsv1-k*, *Rsv1-s*, *Rsv1-h*, *Rsv1-r*, and *Rsv1-n)* has been reported across the germplasm [2,6,7,8]. *Rsv3*, located on chromosome 14, mediates resistance to G5–G7 through a coiled-coil NLR encoded by *Glyma.14G204700* [9], while *Rsv4*, located on chromosome 2, employs an RNase H-mediated antiviral mechanism targeting double-stranded RNA to confer broad-spectrum resistance against all seven strains [10]. Notably, SMV-G1 exhibits the narrowest virulence spectrum, selectively infecting susceptible accessions while failing to establish infection in resistant cultivars, making it an ideal model for dissecting resistance mechanisms [1].

In China, SMV isolates are categorized into 22 strains (SC1–SC22), with resistance governed by the *RSC* (Resistance of SMV in China) locus, encompassing multiple sub-loci such as *RSC3Q*, *RSC3*, *RSC4*, *RSC5*, *RSC7*, *RSC8*, *RSC12*, *RSC14Q*, *RSC18*, and *RSC20*, primarily chromosomes 2, 13, and 14 [4,5]. Several novel resistance loci have been identified on various chromosomes, including 1, 2, 6, 8, 9, 11, 12, 13, 14, 15, and 19 [4,11,12,13,14]. Most SMV resistance loci have been identified through QTL mapping [4,6,9,10,15].

Genome-wide association study (GWAS) and genomic prediction (GP) have accelerated the identification of SMV resistance determinants. For instance, Che et al. [16] identified 24 relevant SNPs associated with strain SC3 resistance, including five novel loci on chromosomes 8 and 12, while Pu et al. [4] mapped *Glyma.15G015700* and six novel loci as key players in SC7 resistance. These identified SNPs and candidate genes have the potential for marker-assisted selection (MAS) in breeding programs aimed at enhancing resistance to SMV. In this investigation, ridge regression best linear unbiased prediction (rr-BLUP) in the rrBLUP package and Bayesian models, including Bayes A (BA), Bayes B (BB), Bayes LASSO (BL), and Bayes ridge regression (BRR), were employed for predicting genomic estimated breeding value (GEBV) in genomic prediction [17].

The *Rsv1* locus in soybeans contains a cluster of nucleotide-binding site/leucine-rich repeat (NBS-LRR) genes that collectively elicit diverse responses to SMV strains [6]. Functional studies implicate defense regulators *GmEDR1*, *GmEDS1*, and *GmHSP90* in *Rsv1*-mediated extreme resistance, while *eIF5A* has been identified as a crucial element in the *Rsv1*-triggered lethal systemic hypersensitive response to SMV-G7 [18,19]. PCR-based markers have been developed for detecting *Rsv1* alleles [20]. The *Rsv1* gene provides resistance to a wide range of SMV strains through an extreme resistance mechanism that does not rely on hypersensitive response (HR) at the inoculation site [21]. The integration of *Rsv1* with *Rsv3* and *Rsv4* in elite lines such as Chinese cultivar 8101 exemplifies the successful deployment of broad-spectrum resistance [22,23,24].

In this study, we performed GWAS and GP analyses on 218 USDA soybean accessions to elucidate the genetic determinants of SMV-G1 resistance. These findings advance marker-assisted breeding strategies and provide insights into the genomic architecture underlying resistance to SMV-G1 in soybean.

## 2. Results

### 2.1. SMV-G1 Resistance Phenotyping

**Figure 1:**
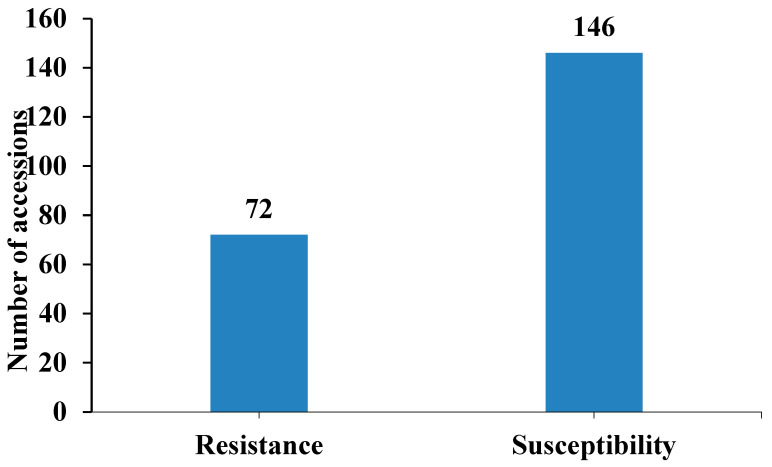
The distribution of soybean mosaic virus reactions among 218 soybean accessions.

Evaluation of 218 soybean accessions revealed 72 resistant (R) (33%) and 146 susceptible (S) (67%) genotypes (Supplementary Table S1, Figure 1). Resistance (R) and susceptibility (S) classifications were maintained consistently across the Supplementary Materials.

### 2.2. Population Structure and GWAS

**Figure 2:**
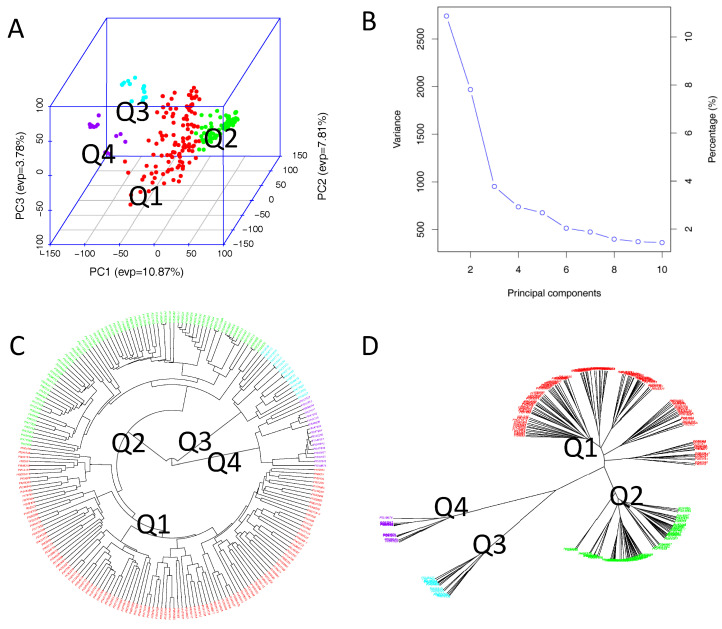
Analysis of penetic diversity in the association panel consisting of 218 USDA soybean accessions: (**A**) Three-dimensional graphical plot of the principal component analysis (PCA). (**B**) PCA eigenvalue plot. (**C**,**D**) Phylogenetic trees ((**C**)—fan, (**D**)—unrooted) drawn using the neighbor-joining (NJ) method in four sub-populations (Q1–Q4) by GAPIT3.

**Figure 3:**
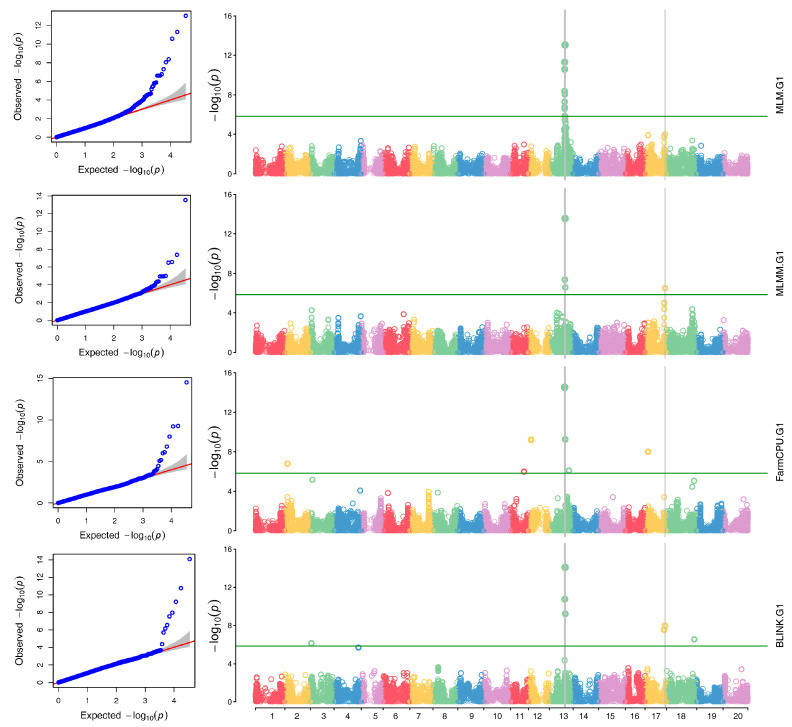
QQ-plots (**left**) and Manhattan plots (**right**) of the results of GWASs on resistance to soybean mosaic virus (SMV) in strain G1 in 218 accessions based on MLM, MLMM, FarmCPU, and BLINK in GAPIT3. In the QQ-plot (**left**), the x-axis is the LOD [−log(*p*-value)] value and the y-axis is the expected LOD [−log(*p*-value)] value. In the Manhattan plot (**right**), the x-axis is the soybean chromosome and the y-axis is the LOD [−log(*p*-value)] value.

Employing GAPIT3, the 218 soybean accessions were categorized into four distinct clusters (subpopulations), designated Q1 to Q4 (Figure 2). This division was based on the following analyses: (1) a 3D graphical plot of the principal component analysis (PCA) (Figure 2A), (2) a PCA eigenvalue plot (Figure 2B), and (3) phylogenetic trees (Figure 2C,D), which were constructed using the neighbor-joining (NJ) method. Additionally, the kinship plot confirmed the existence of these four groups among the 218 accessions (Supplementary Figure S1). Each of the 218 accessions was assigned to one of the four clusters (Q1 to Q4), and the Q-matrix with four matrices was subsequently applied to the GWAS analysis.

Based on the analysis in GAPIT3, the multiple QQ plot distribution showed a significant deviation from the expected distribution (Figure 3, left half; Supplementary Figure S2, right), indicating the presence of SNPs associated with resistance to SMV G1. The multiple Manhattan plot, covering all 34,631 tested SNPs, revealed several SNPs with LOD values greater than 5.84, primarily located on chromosome 13, suggesting the presence of SNPs associated with SMV G1 resistance in the panel (Figure 3, right half; Supplementary Figure S2, left).

Twenty-two SNPs were significantly associated (LOD > 5.84) with G1 resistance across chromosomes 1, 2, 3, 12, 13, 17, and 18 (Figure 3; Supplementary Table S2). Four high-confidence SNPs (LOD > 6.5; detected by ≥ 2 models), Gm13_29459954_ss715614803, Gm13_29751552_ss715614847, Gm13_30293949_ss715614951, and Gm13_30724301_ss715615024, anchored a 1.27 Mb QTL on chromosome 13 (29.46–30.73 Mb), overlapping the Rsv1 locus (Table 1).

### 2.3. Candidate Gene Identification

Thirty-eight genes resided within 5 kb flanking regions of 20 significant SNPs (Supplementary Table S3).

Notably, four disease resistance gene analogs were detected in a region spanning 873 kb, from 29.85 Mb to 30.73 Mb, with strongly associated SNP markers on chromosome 13 (Supplementary Table S3). *Glyma.13G194100*, located from 30,726,074 bp to 30,730,604 bp on chromosome 13, is closely linked to SNP marker Gm13_30724301_ss715615024 (chr 13: 30,724,301 bp) within a distance of less than 2 kb. *Glyma.13G184800* and *Glyma.13G184900* are situated between the two SNP markers, Gm13_29751552_ss715614847 and Gm13_30293949_ss715614951, with distances ranging from 107 kb to 417 kb. Another gene, called *3Gg2* [6,20], is also located in this region between Gm13_30293949_ss715614951 and Gm13_30462695_ss715614981, with distances of approximately 34 kb and 130 kb (Supplementary Table S3).

### 2.4. Genomic Prediction Accuracy

**Figure 4:**
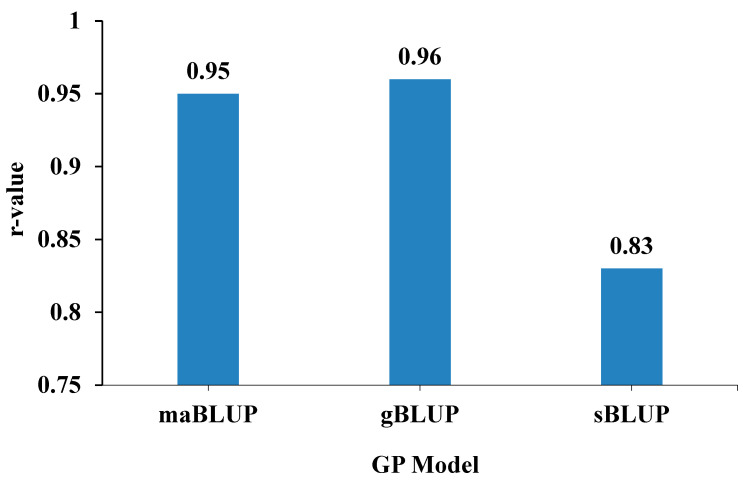
GP model r-value of SMV strain G1 resistance in training sets within a panel of 218 soybean accessions assayed with 34,631 SNPs. The predictions were conducted using three models: maGLUP, gBLUP, and sBLUP in GAPIT3.

*GP in the reference:* The genomic prediction achieved very high accuracy (maBLUP: *r* = 0.95; gBLUP: *r* = 0.96; sBLUP: *r* = 0.83) (Figure 4). These estimates were obtained using a training set to predict SMV G1 resistance in a panel of 218 soybean accessions with all 34,631 SNPs in GAPIT3. This indicates that genomic selection is efficient for selecting SMV G1 resistance.

**Figure 5:**
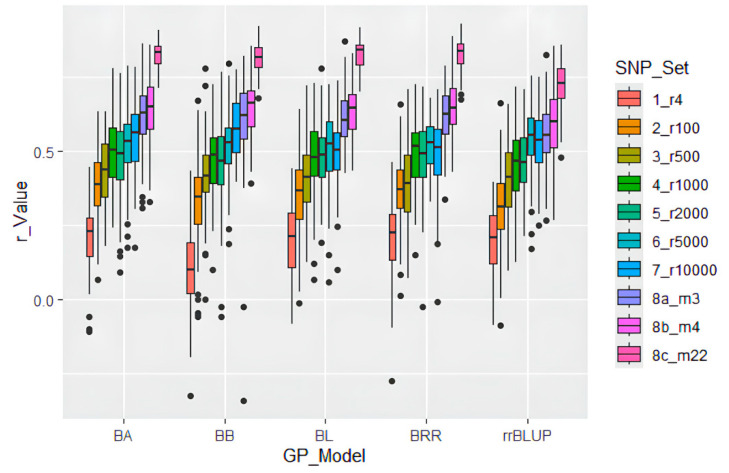
GP model r-value of SMV strain G1 resistance using ten SNP sets: seven random SNP sets, with 4 to 10,000 SNPs, plus three GWAS-derived significant SNP marker sets (3 markers—m3; 4 markers—m4; and 22 markers—m22). Predictions were estimated using five genomic prediction (GP) models: BA, BB, BL, BRR, and rrBLUP.

GP in cross-prediction using randomly selected SNP markers: The genomic prediction via cross-prediction using randomly selected SNP markers showed the following average r-values: 0.19 in r4, ranging from 0.10 to 0.22; 0.35 in r100, ranging from 0.31 to 0.39; 0.41 in r500, ranging from 0.39 to 0.43; 0.48 in r1000, ranging from 0.45 to 0.50; 0.47 in r2000, ranging from 0.46 to 0.48; 0.52 in r5000, ranging from 0.51 to 0.54; and 0.53 in r10000, ranging from 0.49 to 0.58 (Table 2, Figure 5). These results showed that the r-value increased as more randomly selected SNPs were used, from an average of 0.19 in the 4-SNP set to 0.53 in the 10,000-SNP set. This suggests that random SNP sets required ≥ 1000 markers to reach r ≥ 0.48 in genomic selection (GS) of SMV-G1 resistance. GWAS-derived SNP marker sets: The average r-value was 0.60 in m3, ranging from 0.56 to 0.62; 0.63 in m4, ranging from 0.58 to 0.64; and 0.80 in m22, ranging from 0.72 to 0.83. These findings revealed that GWAS-derived SNP markers can be effectively used to estimate GP and to select for SMV-G1 resistance in soybean breeding through MAS and GS (Table 2, Figure 5).

GP Model: Bayesian models (BA, BB, BL, BRR) exhibited comparable efficacy (r ≈ 0.53–0.80), surpassing rrBLUP (r ≈ 0.49–0.58). This suggests that BA, BB, BL, and BRR can be effectively utilized for selecting resistance to SMV-G1 in genomic selection (GS).

### 2.5. Genetic Diversity and Utilization of the SMV-Resistant Accessions

The 72 resistant accessions originated from 15 countries, including China (21 accessions), Japan (13), the United States (12), South Korea (7), North Korea (4), Uruguay (3), Georgia (2), South Africa (2), and 1 each from 7 other countries, plus 1 of unknown origin. The majority (57 accessions; 79.2%) of SMV G1-resistant accessions were from China, Japan, the United States, South Korea, and North Korea (Supplementary Table S4).

**Figure 6:**
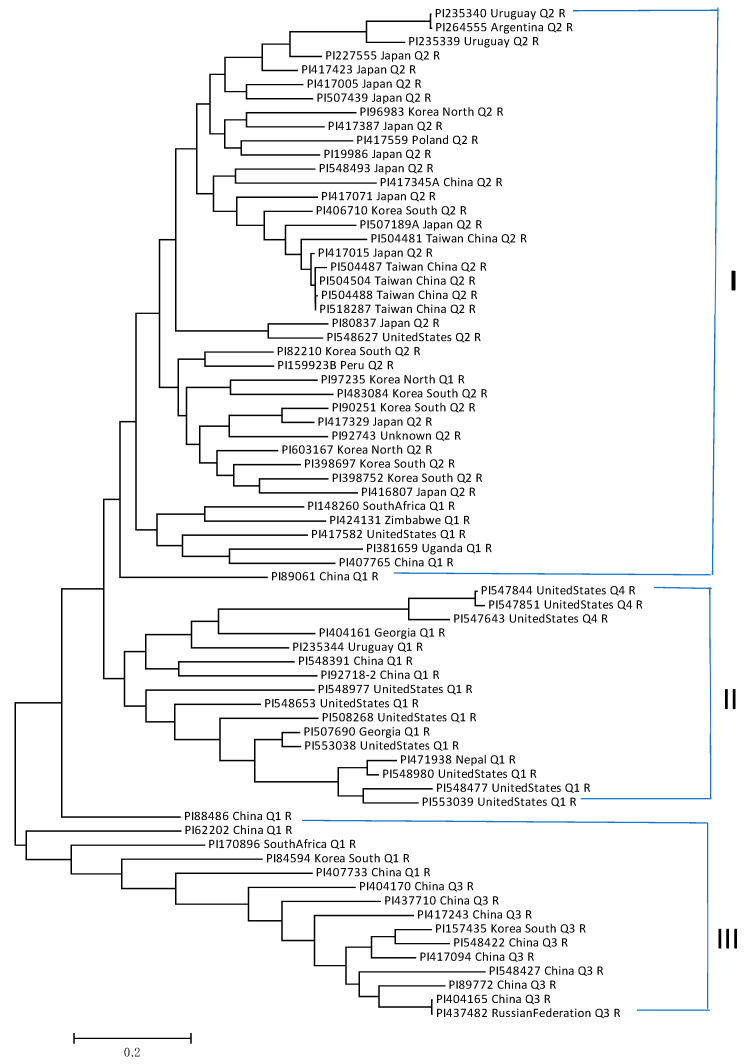
Phylogenetic tree created from MEGA 7 of 72 soybean germplasm accessions resistant to SMV strain G1 using the maximum likelihood (ML) method.

Maximum-likelihood phylogeny resolved three genetic clusters (Figure 6, Supplementary Table S4), as follows.

Group I consists of 41 accessions collected from 12 countries, including Japan (13 accessions), China (8), South Korea (6), North Korea (3), the United States (2), Uruguay (2), and 1 each from 6 other countries, plus 1 of unknown origin. The majority (30 accessions; 73.2%) were from China, Japan, South Korea, and North Korea (Supplementary Table S4b).

Group II consists of 16 accessions collected from five countries: the United States (10), Georgia (2), China (2), Uruguay (1), and Nepal (1). The majority (10 accessions; 62.5%) were from the United States (Supplementary Table S4b).

Group III consists of 15 accessions collected from four countries, including China (11), South Korea (2), the Russian Federation (1), and South Africa (1). The majority (11 accessions; 73.3%) were from China (Supplementary Table S4b).

Geographic analysis showed that all 13 SMV-resistant accessions from Japan were clustered into Group I. Among the 12 accessions from the United States, 10 (83.3%) were classified in Group II. Of the 21 accessions from China, 11 (52.4%) were in Group III, while 8 (19.5%) and 2 (12.5%) were in Groups I and II, respectively (Supplementary Table S4). These findings indicate that geographic factors influence the distribution of SMV-G1-resistant accessions.

Phylogenetic tree analysis of all 218 tested materials showed that 72 G1-resistant materials were located on different branches of the phylogenetic tree using 6000 randomly selected SNPs (Supplementary Figure S3). However, they clustered into several groups when using the four associated SNP markers, Gm13_29459954_ss715614803, Gm13_29751552_ss715614847, Gm13_30293949_ss715614951, and Gm13_30724301_ss715615024 (Supplementary Figure S4). This indicates that accessions with the same G1 resistance gene clustered together, but there were different G1 resistance genes among these accessions.

## 3. Discussion

### 3.1. Phenotypic Evaluation and Genetic Diversity of SMV-G1 Resistance

The screening of 218 soybeans identified 72 accessions (33%) exhibiting resistance to SMV-G1 (Figure 1, Supplementary Tables S1 and S4), indicating that many SMV-G1-resistant accessions were available in the USDA Soybean Germplasm Collections. The 72 resistant accessions were distributed across all branches of the phylogenetic tree of the 218 tested soybean accessions (Supplementary Figure S3). Phylogenetic analysis revealed broad genetic diversity among SMV-resistant accessions, with distinct clustering patterns reflecting geographic origins. Notably, the accessions from China exhibited more variation in genetic backgrounds.

The 72 accessions showed differential reaction patterns for resistance to SMV strains from G1 to G7 in previous studies [2,7,8,20]. Of these, 58 accessions have been reported to carry one gene at the *Rsv1* locus for SMV-G1 resistance (Supplementary Table S5). Further GWAS is ongoing to explore these additional resistance factors across multiple SMV strains.

### 3.2. Genome-Wide Association Study

The GWAS identified four high-confidence SNPs (LOD > 6.5) co-localizing with the *Rsv1* locus on chromosome 13 (29.46–30.73 Mb). Three other SNP markers (Gm13_29751552_ss715614847, Gm13_30293949_ss715614951, and Gm13_30724301_ss715615024) flanked SSR markers (BARCSOYSSR_13_1114, BARCSOYSSR_13_1128, and BARCSOYSSR_13_1155) previously linked to *Rsv1*-mediated resistance [25,26,27]. Yang et al. [26] mapped two Rsv1-associated candidate genes between BARCSOYSSR_13_1128/1136 (SC3, SC6, SC17 resistance) and BARCSOYSSR_13_1140/1155 (SC7 resistance), while Ma et al. [27] delineated *Rsv1-h* within BARCSOYSSR_13_1114/1115. These SSR markers have commonly verified *Rsv1* as the primary SMV G1 resistance locus.

A phylogenetic tree showed that SMV-G1-resistant accessions cluster together in distinct groups, separate from susceptible accessions. Phylogenetic clustering of resistant accessions suggested that the identified SNP markers could be used to verify new resistant accessions (Supplementary Figure S4). However, incomplete separation of resistant and susceptible subgroups indicated that these resistant accessions have likely carried additional resistance genes beyond the *Rsv1* alleles. Further analysis is ongoing to identify additional resistance genes for SMV resistance.

### 3.3. Functional Characterization of Candidate Genes

Four NLR-class genes were prioritized within the 873 kb QTL interval: *Glyma.13G184800*, *Glyma.13G184900*, *Glyma.13G194100*, and *3Gg2*. *Glyma.13G184800*. *Glyma.13G184900* encoded CC-NBS-LRR proteins, one of the largest resistance gene families in plants [28,29]. *Glyma.13G194100* contained an NB-ARC domain for regulating the activity of resistance protein [30]. *3Gg2* was a candidate gene for *Rsv1*, encoding an NBS-LRR similar resistance protein [6,31]. Additionally, using *Rsv1*-f/r, a pair of DNA primers from the *3Gg2* gene sequence, and conducting PCR in 101 soybean genotypes and an F_2_ population segregated at the *Rsv1* locus made it possible to distinguish resistant lines with the *Rsv1* gene from lines with other SMV resistance genes, thereby identifying the *3Gg2* gene at the *Rsv1* locus [20].

In conclusion, the four candidate genes are involved in the translation of plant resistance proteins or the encoding of similar resistance proteins at the *Rsv1* locus, providing a robust tool for marker-assisted pyramiding.

### 3.4. Genomic Prediction for Breeding Applications

Comparative analysis of GP models revealed Bayesian approaches (BA, BB, BL, BRR) All five models exhibited similar high r-values across all of SNP sets and outperformed rrBLUP (r ≈ 0.53–0.80), with three GWAS-derived SNP sets (r ≈ 0.60–0.83) achieving higher accuracy than seven random SNP sets (r ≈ 0.10–0.58) using the same GP model (Table 2; Figure 5).

The scale of SNP sets could also be an important factor influencing GP [32,33]. Through an analysis of seven randomly selected SNP sets ranging from 4 to 10,000 SNPs using five models, we observed that the average r-value decreased significantly with smaller SNP sets. SNP sets with fewer than 2000 randomly selected SNPs may not be suitable for GS related to resistance to SMV strain G1. This finding aligns with Shi et al. [34], who observed similar trends in their study. They used nine randomly selected SNP sets ranging from 9 to 4836 SNPs to conduct cross-prediction in 346 USDA spinach accessions for white rust resistance and observed that the r-value increased from 0.25 to 0.79 as the number of randomly selected SNPs increased from 9 to 4836.

These results suggest that the SMV-G1 resistance in soybean is primarily controlled by major gene(s) or QTLs, indicating that selecting SNPs through GWAS can be highly effective.

## 4. Materials and Methods

### 4.1. Plant Material

A diversity panel of 218 soybean accessions from the USDA Germplasm Collection representing 25 geographic regions, including 1 accession of unknown origin, was used for this study (Supplementary Table S1).

### 4.2. Phenotyping and Genotyping

SMV-G1 resistance data were curated from published screens [7,35] and the USDA GRIN database (https://npgsweb.ars-grin.gov/gringlobal/descriptordetail?id=51159, accessed on 24 February 2024) [7,35]. The accessions were genotyped with Soy50K SNP Infinium Chips [36]. All 36,431 SNPs across 218 soybean accessions were downloaded from SoyBase (https://www.soybase.org/tools/snp50k/, accessed on 24 February 2024) and distributed across all 20 chromosomes of the soybean genome (Supplementary Figure S5) [37].

Virus inoculum was prepared by homogenizing systemically infected leaves from virus-maintaining stock plants in 0.05 M potassium phosphate buffer (pH 7.2) at an approximate dilution of 1:10 (*w*/*v*) using a mortar and pestle. Inoculation was performed by rubbing the inoculum with a pestle onto both unifoliolate leaves that had been previously dusted with carborundum [7,35,38]. Plants of each accession were monitored for SMV symptoms regularly and their reactions were classified according to six types: ER, N, N/R, R, S, and S/R (described above). We used only 218 accessions, consisting of 72 resistant accessions and 146 susceptible accessions, for GWAS and GP in this study.

### 4.3. Principal Component Analysis and Genetic Diversity

Population stratification was assessed via PCA, neighbor-joining phylogeny, and kinship analysis in GAPIT3 [39].

PCA and genetic diversity were analyzed with within R software version 4.3.1 (https://www.r-project.org/, accessed on 1 January 2024) [39], with PCA components set from 2 to 10 and the NJ tree settings from 2 to 10 [39,40]. Phylogenetic trees were drawn using the neighbor-joining method in R package GAPIT3 (GAPIT v.3.1.0).

Genetic diversity was assessed for (1) the 218 accessions and (2) the SMV G1-resistant accessions using 6000 randomly selected SNPs from total 36,431 and using GWAS-derived SNP markers associated with SMV resistance. The phylogenetic trees were generated using MEGA 7 [41] based on the maximum likelihood method with the parameters described in Shi et al. [32,42].

In the multi-model GWAS (MLM, MLMM, FarmCPU, BLINK), a Bonferroni-corrected threshold (*p* < 1.37 × 10^−6^; LOD = 5.84), was employed for significance.

### 4.4. Candidate Gene Annotation

Following the methodology outlined by Zhang et al., candidate genes within 5 kb on both sides of significant SNPs were annotated using Glycine max Wm82.a2.v1 (https://phytozome-next.jgi.doe.gov/info/Gmax_Wm82_a2_v1, accessed on 2 February 2024) [43].

### 4.5. Genomic Prediction

Three methods in the GAPIT3 packages (maGLUP, gBLUP, and sBLUP) were employed to predict GEMV, utilizing all 34,631 SNPs within R software [40]. Then, GEMV prediction was executed using a set of 1000 randomly selected SNPs and two GWAS-derived SNP marker sets (3 and 22 markers—M3 and m22) for the resistance to SMV-G1 in the panel, estimated using four Bayesian models GP models (BA, BB, BL, and BRR) in the BGLR package and rrBLUP in the rrBLUP packages [44,45]. Subsequently, the training and validation sets were randomly created 100 times, and r-value was estimated each time. The average r-value across the 100 iterations was calculated for the SMV-G1 resistance [46]. For both GP scenarios, a higher r-value indicates higher prediction accuracy and better selection efficiency in GS, reflecting the reliability of the genomic predictions for SMV-G1 resistance [47,48].

## 5. Conclusions

This genome-wide association study and genomic prediction analysis using 36,431 SNPs delineates a 1.27 Mb QTL on chromosome 13 harboring four key SNP markers (Gm13_29459954_ss715614803, Gm13_29751552_ss715614847, Gm13_30293949_ss715614951, and Gm13_30724301_ss715615024) and four SMV-G1-resistant candidate genes (*Glyma.13G184800*, *Glyma.13G184900*, *Glyma.13G194100*, and *3Gg2*) underlying SMV-G1 resistance using 218 soybean germplasm accessions from the USDA Germplasm Collection. High genomic prediction accuracy was observed using three GWAS-derived SNP sets. This study provides valuable gene information for selecting SMV-G1-resistant plants and lines in soybean breeding and enables precision pyramiding of *Rsv1* alleles with complementary resistance loci to accelerate the development of durable SMV-G1-resistant cultivars.

## Figures and Tables

**Table 1:** List of four SNPs with LOD [−log(*p*-value)] greater than 5.84 detected by two or more models (BLINK, FarmCPU, MLMM, or MLM) in GAPIT3 and their position on chromosomes based on the soybean genome assembly Wm82.a2.v1.

SNP	Chr	Position	LOD [−log(*p*-Value)]	MAF%	Model
Gm13_29459954_ss715614803	13	29459954	10.58	19.5	MLM
			14.53	19.5	FarmCPU
Gm13_29751552_ss715614847	13	29751552	8.06	44	MLM
			7.37	44	MLMM
			10.76	44	BLINK
Gm13_30293949_ss715614951	13	30293949	13.04	13.5	MLM
			13.54	13.5	MLMM
			14.09	13.5	BLINK
Gm13_30724301_ss715615024	13	30724301	6.56	22.48	MLMM
			9.21	22.48	BLINK

**Table 2:** GP model r-value and standardized errors of the r-values (SE) of SMV strain G1 resistance using ten SNP sets: seven random SNP sets with SNP number from 4 to 10,000 and three GWAS-derived SNP marker sets (3 markers—m3; 4 markers—m4; and 22 markers—m22). Predictions were performed using five GP models: BA, BB, BL, BRR, and rrBLUP.

SNP Set	GP Model r-Value						SE					
	BA	BB	BL	BRR	rrBLUP	SNP Set Mean	BA	BB	BL	BRR	rrBLUP	SNP Set Mean
r4	0.22	0.10	0.20	0.21	0.20	0.19	0.011	0.013	0.012	0.012	0.011	0.012
r100	0.39	0.33	0.35	0.37	0.31	0.35	0.012	0.014	0.014	0.011	0.013	0.013
r500	0.43	0.42	0.41	0.39	0.40	0.41	0.010	0.011	0.012	0.013	0.012	0.011
r1000	0.50	0.47	0.48	0.50	0.45	0.48	0.011	0.011	0.012	0.011	0.012	0.012
r2000	0.48	0.46	0.48	0.48	0.46	0.47	0.012	0.013	0.010	0.012	0.011	0.012
r5000	0.52	0.51	0.51	0.52	0.54	0.52	0.012	0.010	0.012	0.009	0.011	0.011
r10000	0.54	0.58	0.50	0.49	0.53	0.53	0.011	0.010	0.011	0.013	0.010	0.011
m3	0.61	0.60	0.61	0.62	0.56	0.60	0.011	0.015	0.009	0.009	0.011	0.011
m4	0.64	0.64	0.63	0.64	0.58	0.63	0.011	0.009	0.008	0.009	0.012	0.010
m22	0.83	0.81	0.82	0.82	0.72	0.80	0.004	0.005	0.005	0.005	0.007	0.005
Model Mean	0.52	0.49	0.50	0.51	0.48	0.50	0.010	0.011	0.010	0.010	0.011	0.011

## Data Availability

Phenotype data of 218 soybean accessions can be accessed through the USDA GRIN website (https://npgsweb.ars-grin.gov/gringlobal/descriptordetail?id=51159, accessed on 24 February 2024). Genotype data of 36,431 SNPs can be accessed through SoyBase (https://www.soybase.org/tools/snp50k/, accessed on 24 February 2024). Phenotype data are available in Supplementary Table S1. Imputed genotype data are available in Supplementary Table S2. Supplementary Table S6 lists 36,431 SNP alleles, chromosome, positions, and genotypes for different soybean accessions.

## Supplementary Materials

The following supporting information can be downloaded at: https://www.mdpi.com/article/10.3390/ijms26052106/s1.
