# Supplementary material for: Genome-Wide Association Study and Genomic Prediction of Soybean Mosaic Virus Resistance
Source: Int J Mol Sci. 2025 Feb 27;26(5):2106. doi: 10.3390/ijms26052106 (PMC11900104; doi:10.3390/ijms26052106)
Supplement: Supplementary file 1 [file ijms-26-02106-s001.zip › Supplimental Information ú¿Figure S1-S5ú⌐.pdf]

Supplementary Information

Supplemental Figures S1 - S5

Article Title: Genome-Wide Association Study and Genomic Prediction of Soybean Mosaic Virus Resistance

Di He[1,2,†], Xintong Wu[1,†], Zhi Liu[1], Qing Yang[1], Xiaolei Shi[1], Qijian Song[3], Ainong Shi[4], Dexiao Li[5,*], Long Yan[1,2,*]

[1] Institute of Cereal and Oil Crops, Hebei Academy of Agricultural and Forestry Sciences, Shijiazhuang 050035, China; ternuraeyes@163.com (D.H.); xintongwu66@163.com (X.W.); zhiliulin@sina.com (Z.L.); qyang0807008@163.com (Q.Y.); shixiaolei59@163.com (X.S.)

[2] College of Life Sciences, Hebei Agricultural University, Baoding 071001, Hebei, China

[3] Soybean Genomics and Improvement Laboratory, Agricultural Research Service, Beltsville, MD 20705, USA; qijian.song@usda.gov

[4] Department of Horticulture, University of Arkansas, Fayetteville, AR 72701, USA; ashi@uark.edu

[5] College of Agronomy, Northwest A&F University, Yangling 712100, China

[*] Correspondence: lidexiao@nwsuaf.edu.cn (D.L.); dragonyan1979@163.com (L.Y.)

[†] These authors contributed equally to this work.

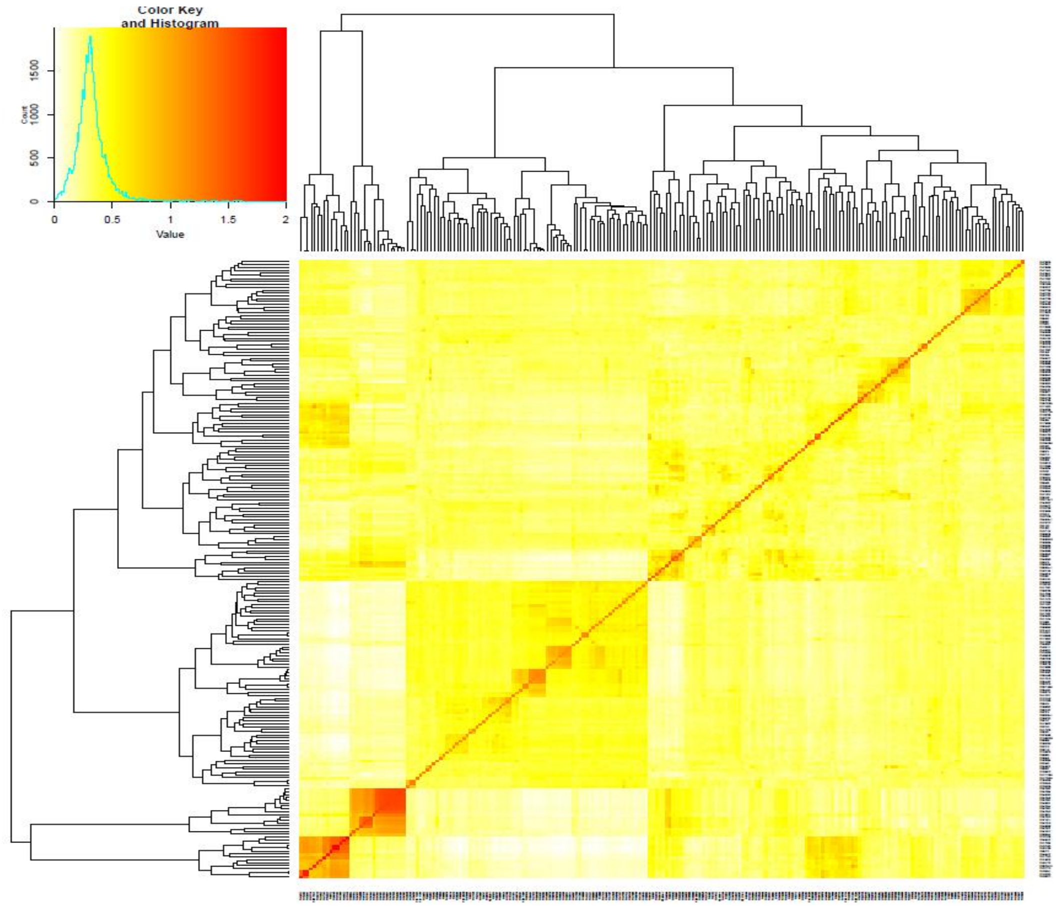

Supplementary Figure S1. Kinship plot drawn by GAPIT3 in 218 soybean accessions, showing four distinct groups.

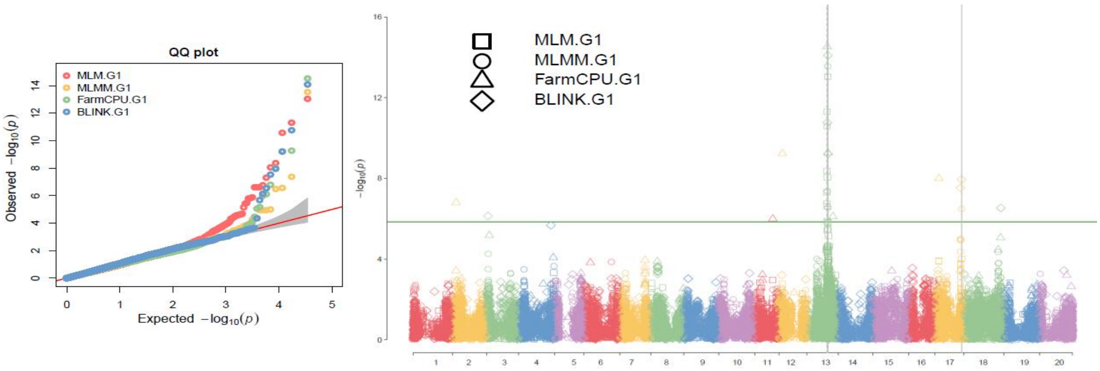

Supplementary Figure S2. QQ plot (**left**) and Multiple Manhattan plot (**right**) comparing symphysic MLM, MLMM, FarmCPU, and BLINK models in GAPIT3 for resistance to SMV strain G1 in an association panel consisting of 218 accessions: The Manhattan plot (**right**) illustrates soybean 20 chromosomes on the x-axis and LOD [−log(*p*-value)] values on the y-axis. The QQ plot (**left**) displays LOD [−log(*p*-value)] values on the x-axis and expected LOD [−log(*p*-value)] values on the y-axis.

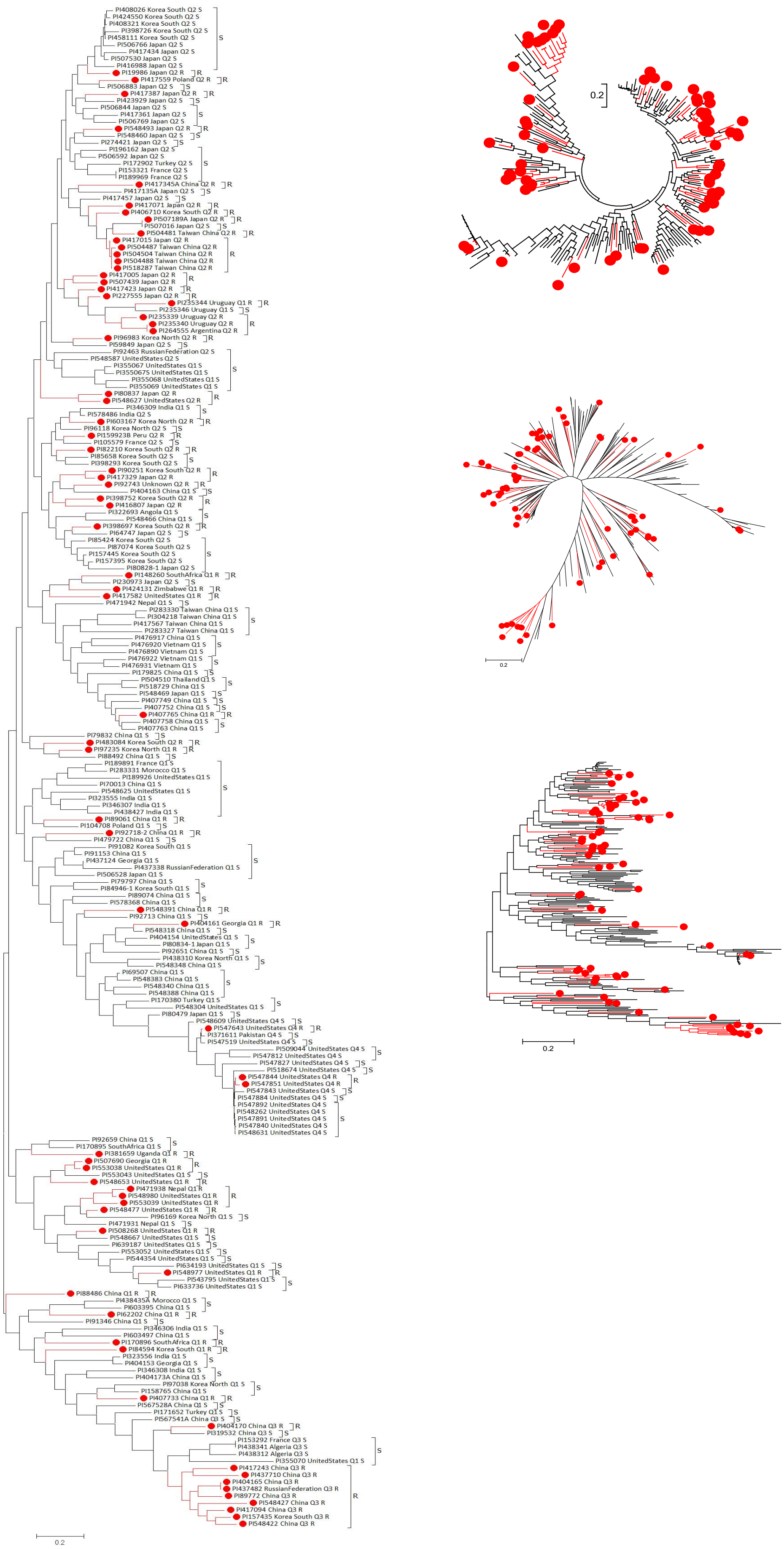

Supplementary Figure S3. Phylogenetic tree generated by ML method using MEGA 7, based on 6,000 SNPs randomly selected from 34,631 SNPs in 218 soybean accessions. In this tree drawn by MEGA 7, the soybean accession number, original country, population structure (clusters Q1 to Q4), and the SMV G1 resistance (R and S) were merged into one taxon name for each soybean accession ID. The red colored shapes and branches represent the resistant accessions.

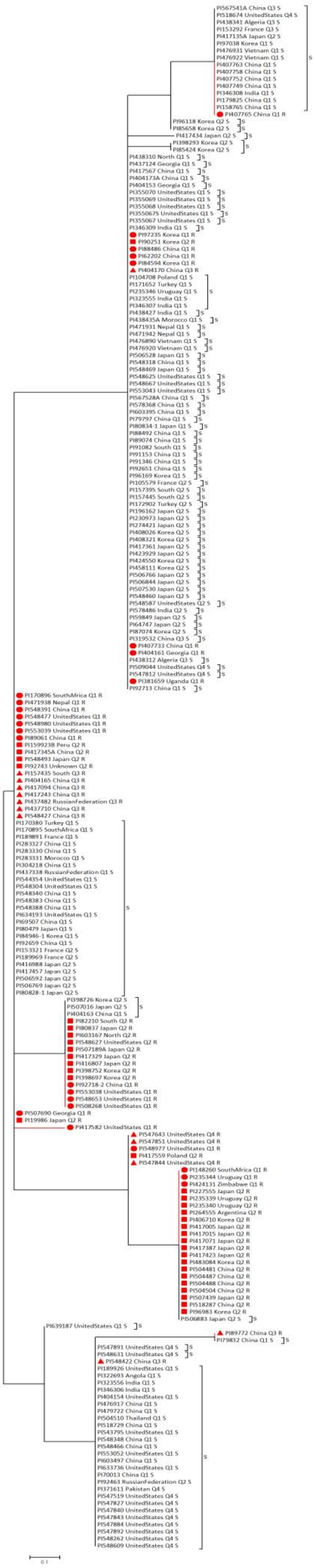

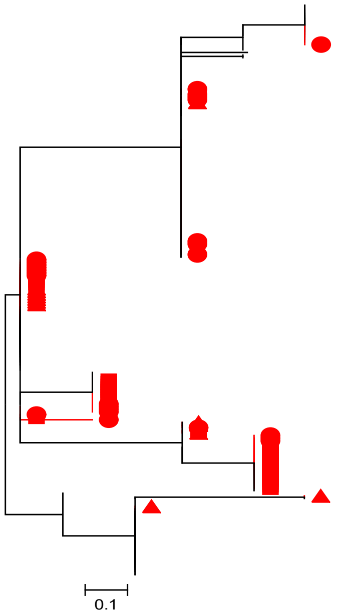
0.1

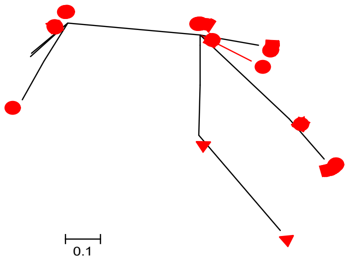
0.1

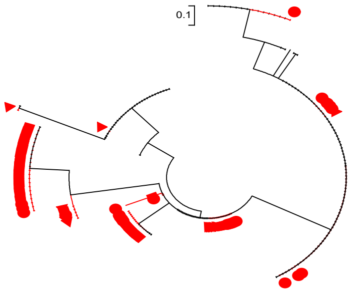
0.1

Supplementary Figure S4. Phylogenetic tree generated by ML method using MEGA 7, based on four SNP markers (listed in Table 1) associated with the resistance to SMV strain G1 in 218 soybean accessions. In this tree drawn by MEGA 7, the soybean accession number, original country, population structure (clusters Q1 to Q4), and the SMV G1 resistance (R and S) were merged into one taxon name for each soybean accession ID. The red colored shapes and branches represent the resistant accessions.

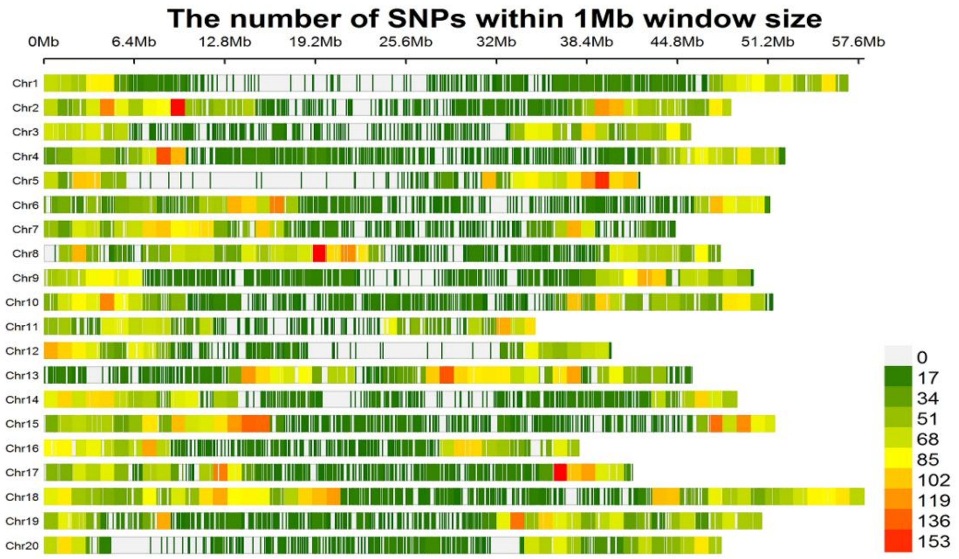

Supplementary Figure S5. Distribution of the 34,631 SNPs on the 20 chromosomes of soybean. Soybean chromosomes are on the vertical axis. Chromosome length in Mb is on the horizontal axis, and the color represents the number of SNPs per 1 Mb window size, SNP density.
